# Immune classifier-based signatures provide good prognostic stratification and predict the clinical benefits of immune-based therapies for hepatocellular carcinoma

**DOI:** 10.1186/s12935-021-02183-5

**Published:** 2021-09-06

**Authors:** Chen Xue, Xinyu Gu, Lanjuan Li

**Affiliations:** State Key Laboratory for Diagnosis and Treatment of Infectious Diseases, National Clinical Research Center for Infectious Diseases, Collaborative Innovation Center for Diagnosis and Treatment of Infectious Diseases, The First Affiliated Hospital, College of Medicine, Zhejiang University, No. 79 Qingchun Road, Shangcheng District, Hangzhou, 310003 Zhejiang China

**Keywords:** Hepatocellular carcinoma, Immune purity, Immune infiltration, Classification, Prognostic model

## Abstract

**Background:**

Hepatocellular carcinoma (HCC) is an aggressive cancer with a high rate of death globally. The use of bioinformatics may help to identify immune cell-related genes both as targets for potential immunotherapies and for their value associated with predicting therapy responses.

**Methods:**

In this study, mRNA expression profiles of HCC samples from The Cancer Genome Atlas (TCGA) database were subjected to gene enrichment, cell type abundance, immune cell infiltration, and pathway enrichment analyses to determine immune cell gene features, cell type abundance, and functional annotation characteristics. We also evaluated their prognostic values using Cox regression and Kaplan–Meier analyses and assessed potential responses to chemotherapy. Four subgroups (Groups 1–4) were identified. Group 4 was associated with advanced clinical characteristics, high immune cell enrichment scores, and the poorest outcomes.

**Results:**

Differentially expressed genes (DEGs) in the HCC samples were enriched in the following pathways: antigen binding, cell surface receptor signal transduction of the immune response, and cell surface activated receptor signal transduction of the immune response. Highly expressed genes in Group 4 were enriched in elements of the WNT signalling pathway. We identified five immune-related genes (*SEMA3A, TNFRSF11B, GUCA2A, SAA1*, and *CALCR*) that were significantly related to HCC prognosis. A prognostic model based on these five genes exhibited good predictive value, with 1-year and 5-year area under the curve (AUC) values of  >  0.66. Group 4 was also potentially more sensitive to EHT 1864, FH535, and lapatinib chemotherapies than the other groups.

**Conclusions:**

We identified and validated four HCC subgroups based on immune system-related genes and identified five genes that may be used for an immune-based prognostic model for HCC treatment.

**Supplementary Information:**

The online version contains supplementary material available at 10.1186/s12935-021-02183-5.

## Background

Liver cancer ranks fifth in neoplasm frequency and has the second highest rate of cancer-associated mortality worldwide [1, 2]. Hepatocellular carcinoma (HCC) accounts for 80% of primary liver cancers and 90% of non-metastatic liver tumours [3–5], with approximately 8,54,000 new cases and 8,10,000 deaths per year globally [6, 7]. The pathology of HCC is multifactorial and involves many steps [8]. It has been reported that viral hepatitis infection, aflatoxins, alcohol, altered transcriptional regulation, and genetic susceptibility/polymorphisms are all considered significant factors (individually or synergistically) that contribute to the aetiology of HCC. The poor prognosis of HCC is mainly due to a lack of sensitive detection methods at its early stages and a high frequency of both recurrence and metastasis [9]. Currently, resection surgery, transplantation, targeted chemotherapy, radiation therapy, interventional therapy, and gene therapy are all effective alternative options for HCC [10, 11]. Although other advanced treatments have been explored, local and regional therapies are still recommended for early-stage disease because few effective options exist for advanced-stage HCC. Recently, however, immune system-associated therapies have been successfully tested in the clinic.

Tumour immunotherapy has revolutionized cancer treatments and has consistently been the focus of attention because of its promising outcomes for advanced HCC [12, 13]. Active and passive immunotherapies, immune checkpoint inhibitors (ICIs), and therapies targeting the tumour microenvironment constitute major breakthroughs for cancer treatments [13, 14]. Accumulating evidence has demonstrated that ICIs [e.g., those targeting programmed cell death ligand 1 (PD-1), T cell immunoglobulin mucin domain-containing-3 (TIM3), and cytotoxic T lymphocyte antigen 4 (CTLA4)] in combination with conventional therapies exhibited enhanced anti-tumour effects and broad applicability for cancer patients [15–17]. Despite these considerable achievements, questions remain as to how to improve the efficacy of immunotherapies, how to broaden their application range, and how to better predict immune responses [17, 18]. Immune system-based models can provide detailed mechanistic insights and be used to categorize patients into low, medium, and high immune response subgroups.

Bioinformatic analyses allow the identification of potential immune-sensitive therapeutic biomarkers, prognostic models can be constructed based on immune system-related genes [19, 20]. In this study, we performed a comprehensive analysis of HCC-related immune infiltration and constructed a gene-based immune response model for predicting immunotherapy responses and for identifying potential biomarkers for HCC-targeting therapies.

## Methods

### Cell culture

The normal human liver cell line L02 and the HCC cell line Hep3B were purchased from the Chinese Academy of Sciences (Shanghai, China) and cultured in DMEM (Gibco, Carlsbad, CA, USA) supplemented with 10% foetal bovine serum and 1% penicillin. Cells were cultured at 37 °C in a 5% CO_2_ atmosphere.

### Quantitative reverse-transcription PCR (qRT-PCR)

Total RNA was extracted from cells using an RNeasy Mini Kit (Qiagen, Valencia, USA) and then reverse transcribed into cDNA using the PrimeScript™ RT reagent Kit according to the manufacturer’s instructions. Relative mRNA expression levels were determined on an ABI 7500 Fast PCR instrument. GAPDH was used as the internal control. Relative expression levels of *IL6, CCR3, SAA1*, and *GCG* were quantified using the 2^−ΔΔCt^ method. The primers are listed in Additional file 1: Table S1.

### Public databases

We collected gene expression profile data (RNA-seq) from HCC patient samples in The Cancer Genome Atlas (TCGA) database (https://cancergenome.nih.gov/). In total, data from 371 patients with follow-up, status, and gene expression data were included in the liver hepatocellular carcinoma (LIHC) cohort. An additional 212 HCC datasets were downloaded from the International Cancer Genome Consortium (ICGC-LIRI-JP, https://dcc.icgc.org/projects/LIRI-JP) for further validation. The ICGC-LIRI-JP dataset was used to verify the prognosis-related immune biomarker genes in the HCC cohort.

### Identification of immune cell-associated genes

We downloaded immune cell-related gene profiles from the ImmPort database [21] (http://www.immport.org). Immune cell-related genes were classified into different subgroups based on different immune cell characteristics, including cell type, functions, and associated pathways [22].

### Single-sample gene set enrichment analysis (ssGSEA)

To evaluate immune cell infiltration characteristics in HCC patient samples, we performed ssGSEA to evaluate the degree of immune cell enrichment in the different samples. ssGSEA was performed using the gene set variation analysis (GSVA) package in R software [23].

### Assessments of immune scores and stromal scores and identification of differentially expressed genes (DEGs)

We utilized the Estimation of STromal and Immune cells in MAlignant Tumour tissues using Expression data (ESTIMATE) method [24], as well as the CIBERSORT tool [25] and other algorithms to estimate cell type abundance from bulk tissue transcriptomes and to assess tumour immune scores and immune cell purities. The Microenvironment Cell Populations-counter (MCP-counter) [26], accessed through Connectivity Map at https://portals.broadinstitute.org/cmap/, was used to predict potential drug sensitivities. In addition, the DESeq package [27] in R software was applied to determine differential gene expression in HCC tumour tissue and adjacent normal tissue from the raw CGA datasets. We defined the false discovery rate (FDR) to be no more than 0.05 and the |log_2_ (fold change)| to be no less than one as the cut-off value.

### Pathway enrichment analysis

To identify immune cell-related pathways, we utilized Kyoto Encyclopedia of Genes and Genomes (KEGG) and Gene Ontology (GO) analyses. Biological process (BP), molecular function (MF), and cellular component (CC) pathway analyses were included in the GO analysis. These analyses were performed using the clusterProfiler package in R software [28, 29].

### Protein–protein interaction (PPI) analysis

To investigate the key regulatory genes and validate the connections between differentially expressed genes, a PPI network was constructed from gene expression datasets from the HCC cohort. We constructed the PPI network based on known and predicted PPIs. Furthermore, we defined the confidence score as  ≥  0.7, and the maximum number of interactors was identified as 0. These selected genes were input into Cytoscape (version 3.6.1) under the following parameters: degree cut-off  =  2, haircut on, node score cut-off  =  0.2, k-core  =  2, and max. depth  =  100.

### Cox regression analysis

We used single-factor Cox regression analysis to identify prognosis-related genes and to assess the effects of the immune status on patient prognoses [30]. We set a *p *value  <  0.01 as the significance level for immune cell-related genes. Multivariate Cox analysis was performed to calculate the risk scores. The median was defined as the dividing line between the high and low scores.

### Evaluation of responses to clinical immunotherapies

Both a submap and the Tumour Immune Dysfunction and Exclusion (TIDE) algorithm were used to predict potential responses to ICIs [31].

### Chemotherapy response predictions

A dataset from the Genomics of Drug Sensitivity in Cancer (GDSC, https://www.cancerrxgene.org/) [32] database was used to screen for potential chemotherapeutics for the treatment of HCC. We estimated the half-maximal inhibitory concentrations (IC50s) for these drugs using the pRRophetic package in R software.

### Statistical analysis

All statistical analyses were performed using SPSS 25 (IBM Corporation, Armonk NY) and R software (version 3.6.1). All statistical results with p  <  0.05 were considered significant.

## Results

### Immune cell-related gene selection, scoring, clustering, and classification

**Fig. 1:**
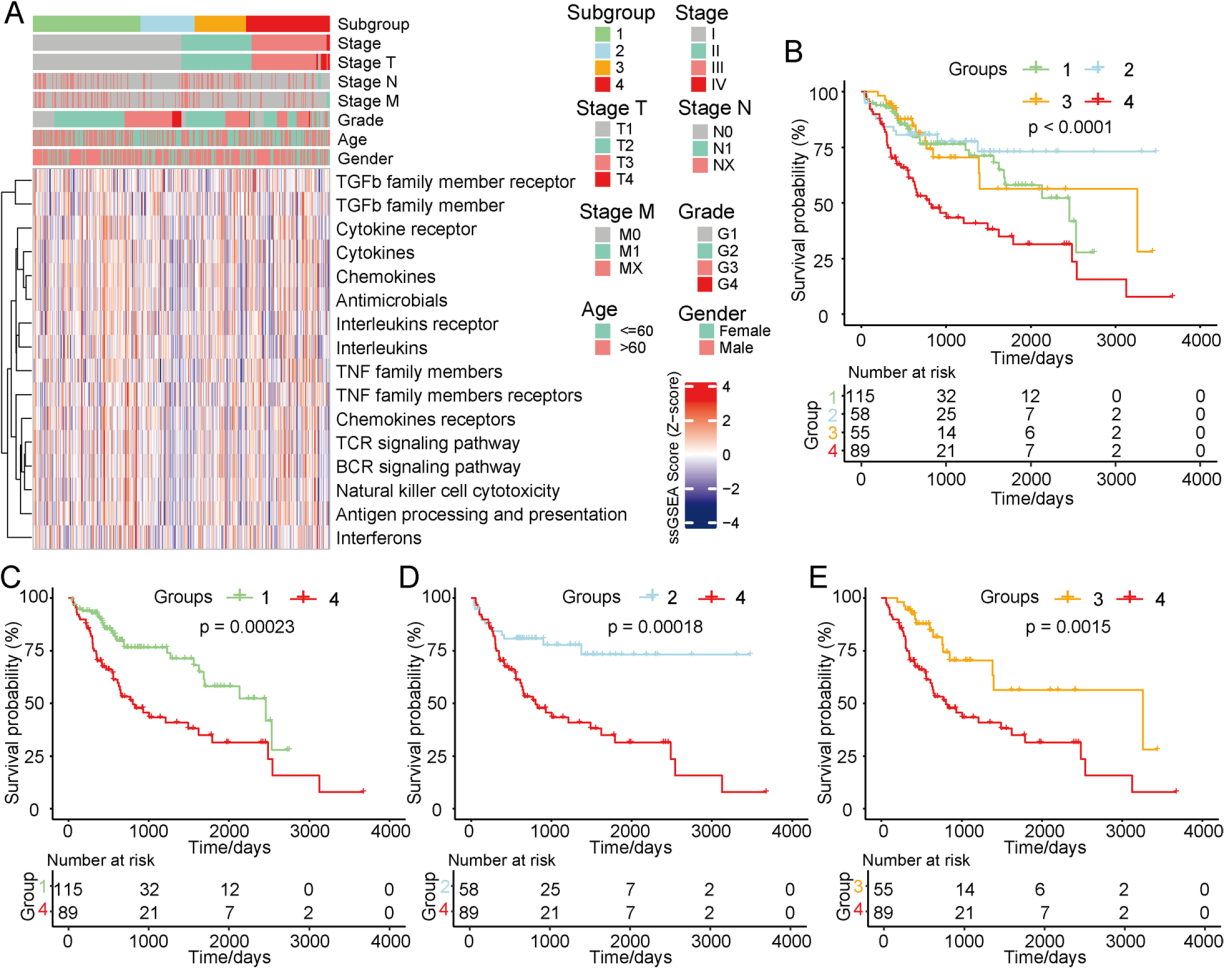
ssGSEA based on the immune system-related gene classifier and prognostic analyses. **a** Immune system-related gene clustering and classification of Groups 1–4. **b** Overall survival analyses of the four groups, with Group 4 showing the poorest prognoses. **c** Prognostic analysis comparing Group 1 to Group 4, showing that Group 1 had better prognoses than Group 4. **d** Group 2 also demonstrated longer overall survival times than Group 4. **e** Group 3 also demonstrated better overall prognoses than Group 4

To construct an immune cell gene-based classifier, we performed ssGSEA and consistent cluster analysis. Based on the different gene scores, we obtained four immune cell gene subgroups (Groups 1–4) (Fig. 1A). Advanced-stage HCC was mainly found in Group 4. A total of 16 immune cell-related gene clusters with high scores were also clustered in Group 4, corresponding with the activated immune subgroups. To further explore the prognoses associated with these four different subgroups, we conducted Kaplan–Meier analysis of overall survival, and the results demonstrated that the prognoses of patients in Group 4 were worse than those of patients in the other groups (Fig. 1B). Specifically, the prognoses of patients in Group 1 were better than those of patients in Group 4 (*p * =  0.00023) (Fig. 1D); Group 2 had more favourable overall survival times than Group 4 *(p * =  0.00018) (Fig. 1D); and the prognoses of patients in Group 3 were better than those of patients in Group 4 (*p*  =  0.0015) (Fig. 1E). We identified four HCC sample subgroups based on the immune scores, and Group 4 was associated with the most advanced clinical stages and significantly poorer prognoses than the other groups.

### Immune cell infiltration in the different subgroups

**Fig. 2:**
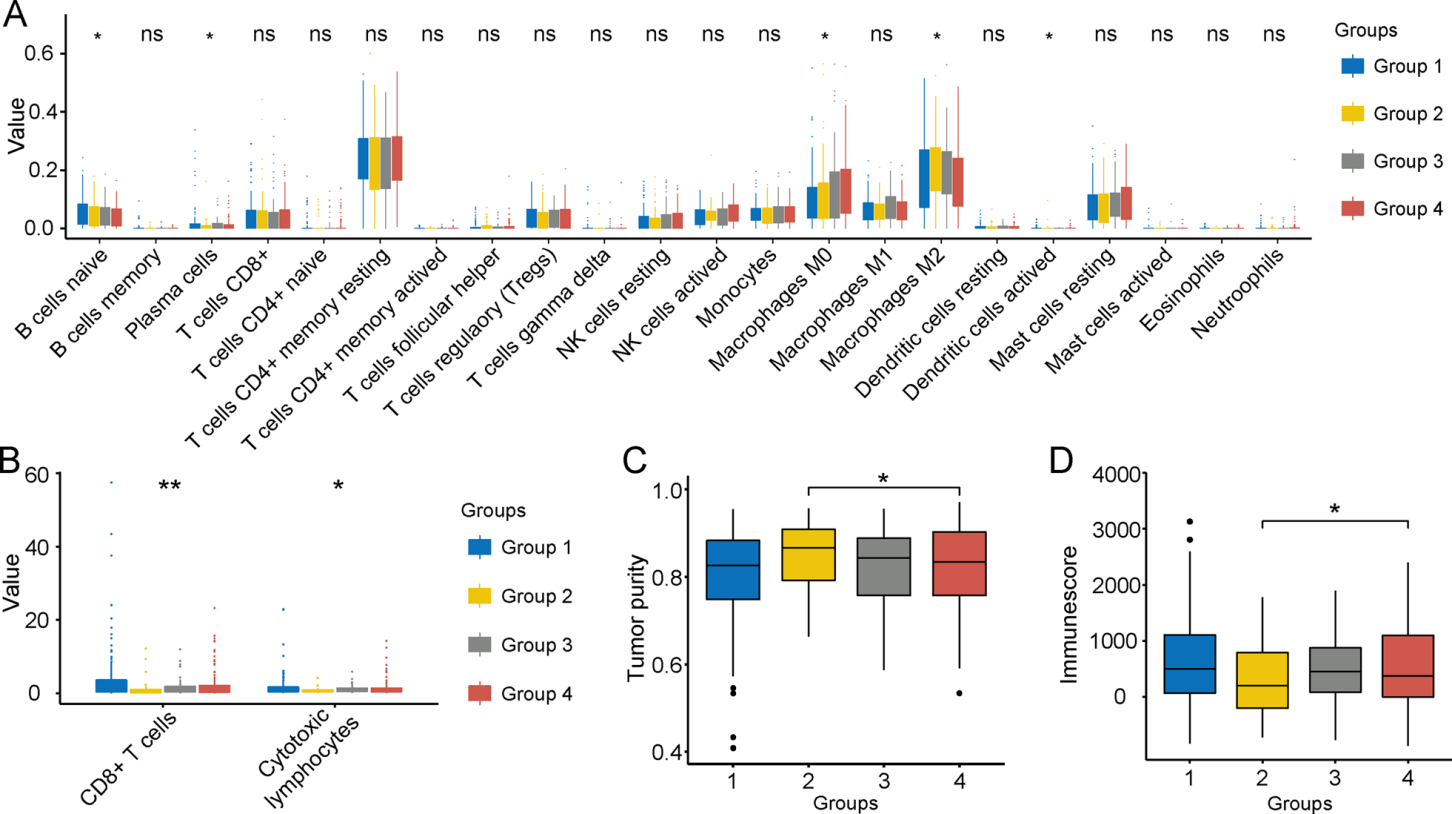
Immune cell infiltration and differences among the four immune-based subgroups. **a** Immune cell infiltration levels in the four subgroups. **b** The levels of CD8^+^ T cells and cytotoxic lymphocytes were significantly different among the four groups. Group 1 had the highest levels of CD8^+^ T cell and cytotoxic lymphocyte infiltration. **c** ESTIMATE analysis indicated that Group 2 had the highest immune purity. Group 2 also had significantly higher immune purity than Group 4. **d** The immune scores demonstrated that Group 2 had the lowest immune scores and were significantly different from those of Group 4

To further investigate immune cell infiltration in the four subgroups, we applied the CIBERSORT algorithm to the data and confirmed that the different subgroups also had significantly different levels of immune cell infiltration. There were significant differences in the proportions of naïve B cells, plasma cells, M0 macrophages, and resting dendritic cells. Group 4 had the lowest proportions of naïve B cells, plasma cells, and resting dendritic cells but the highest proportion of macrophages (Fig. 2A). The MCP-counter algorithm was used to assess the infiltration levels of immune cells in the HCC samples. The results showed that the infiltration levels of CD8^+^ T cells and cytotoxic lymphocytes were significantly different. Group 1 had higher degrees of CD8^+^ T cell and cytotoxic lymphocyte infiltration than the other groups (Fig. 2B). We also used the ESTIMATE method and found that Group 2 had the highest immune purity, with significant differences from that of Group 4 (Fig. 2C). Group 2 also had the lowest immune scores, and these scores were significantly different than those of Group 4 (Fig. 2D). Overall, the four-group classifier of immune system-related genes showed significant differences in immune scores, immune cell infiltration levels, and immune-based prognoses. Group 4 had higher levels of immune cell infiltration, was associated with advanced HCC stages, and had shorter overall survival times. Therefore, tumour immune escape might occur in samples from Group 4, with high immune infiltration, representing disabled and exhausted immune functions.

### Correlations between the immune-based classifier and human leukocyte antigen (HLA) and interactions with immune checkpoint molecules

**Fig. 3:**
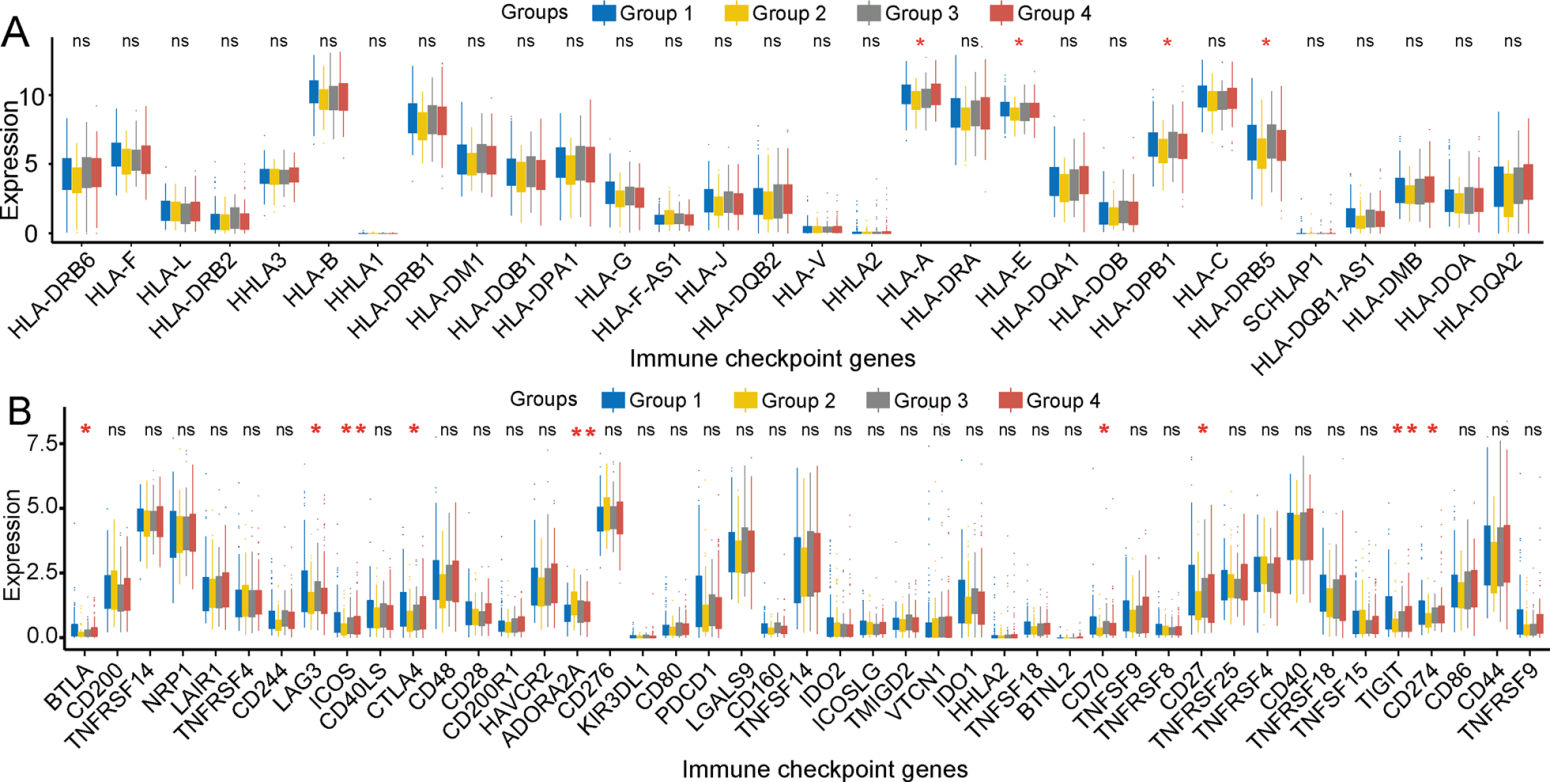
Correlations between the immunophenotype and expression of HLA genes and immune checkpoint molecules. **a** HLA genes (HLA-A, HLA-E, HLA-DPB1, and HLA-DRB5) were differentially expressed in the different groups. **b** Immune checkpoint genes, including *BTLA*, *LAG3*, *ICOS*, *ADORA2A*, *CD70*, *PD-L1* (*CD274*), and *TIGIT,* were differentially expressed in the different groups

Immune checkpoint blockade (ICB) has been widely applied to treat a variety of tumours and shown significant favourable therapeutic effects [33]. HLA molecules are crucial for immune system function and are very clinically significant in immunotherapy [34]. The functional diversity of HLA genes is closely related to cancer genomics and cancer progression [35]. Importantly, responses to ICIs rely on the evolved efficiency of HLA-mediated immunity [36, 37]. We next investigated the internal relationship between the four groups of immune subgroups and HLA genes and identified several immune regulatory molecules, including HLA-A, HLA-E, and HLA-DRB5 (Fig. 3A). To further support correlations between immune checkpoint molecules and our immune-based classifier, we also determined that CD244, ICOS, ADORA2A, CD70, PD-L1 (CD274), and TIGIT molecules showed significant differences among the different immune-based subgroups (Fig. 3B). These results indicate that HLA genes and immune checkpoint molecules play crucial roles in the different subgroups and that they may be valid therapeutic targets for cancer therapies.

### Correlations between immune subgroups and interferon-γ (IFN-γ) pathways

**Fig. 4:**
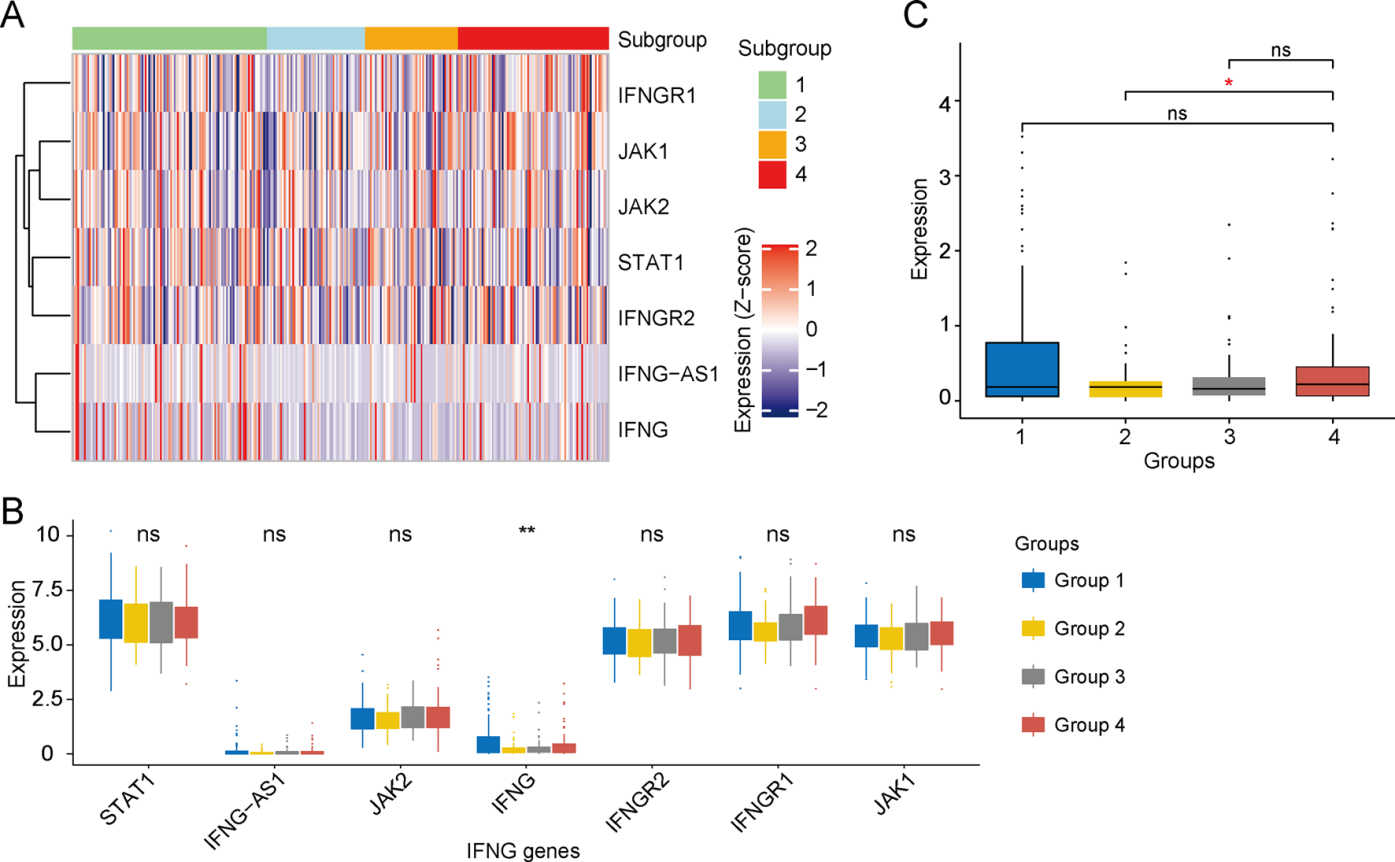
Associations between the immunophenotype and IFN-γ pathways. **a** Correlations between immune responses and IFN-γ pathway-related genes. The *IFN-γ, IFNGR2, IFNGR1, JAK1*, and *JAK* genes were highly expressed in Group 4. **b** There were significant differences in IFN-γ expression among the four subgroups. **c** The expression level of IFN-γ in Group 4 was significantly higher than that in Group 2

The tumour microenvironment is crucial for tumourigenesis and tumour development, and the CD8^+^ T cell-mediated anti-tumour response has been shown to be significantly increased through the production of cytokines such as INF-γ [38]. Accumulating evidence has shown that immune cells, such as CD8^+^ T cells, can facilitate the upregulation of immune checkpoints and enhance anti-tumour immune responses [39]. To annotate IFN-γ and T helper cell-related genes, we performed cluster analysis and found that the immune response has a crucial relationship with IFN-γ-related regulatory pathways (Fig. 4A). More importantly, Group 4 had significantly high expression levels of IFN-r-, IFNGR2-, IFNGR1-, JAK1-, and JAK2-related genes, with IFNG showing significant differences among the subgroups (Fig. 4B). Group 4 had higher IFNG expression levels than Group 2 (Fig. 4C).

### Relationship between the immune subgroups and 5-methylcytosine (m^5^C) regulators

**Fig. 5:**
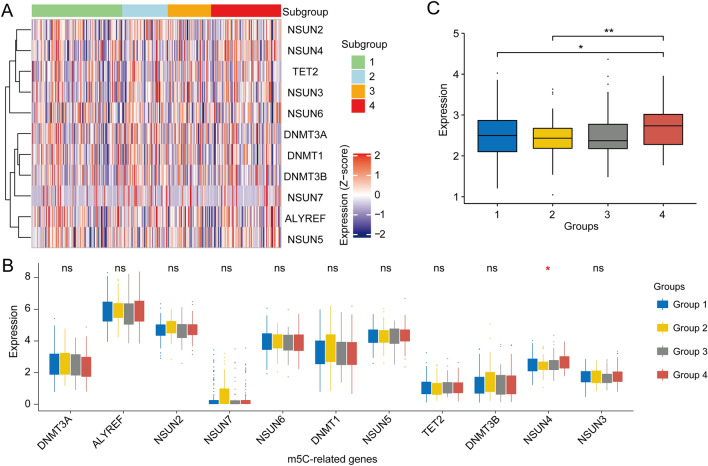
Relationship between the immune system-based classifier and the expression of m^5^C mRNA methylation regulators. **a** All four groups exhibited significant m^5^C regulator activities. **b**, **c** mRNA expression of the m^5^C regulator NSUN4 was significantly different among the four groups, with Group 4 having higher NSUN4 expression than Groups 1 and 2

mRNA and its transcription play an important role in immune-related gene regulation [40, 41]. The m^5^C methylation of RNA modifies the transcription of multiple genes, regulates protein expression, and affects cell phenotypes, and nucleoside modifications have been shown to suppress the potential for RNA to activate innate immune cells [42]. Here, m^5^C regulators were associated with all four groups. The NSUN7 regulator was expressed at low levels in all of these subgroups (Fig. 5B), and the overall levels of m^5^C regulator expression showed obvious differences between Group 2 and Group 4 (*p*  <  0.01) and less obvious differences between Group 1 and Group 4 (*p * <  0.05) (Fig. 5B). The mRNA expression of NSUN4 was remarkably different among the immune subgroups (Fig. 5C).

### Functional annotations of the DEGs

**Fig. 6:**
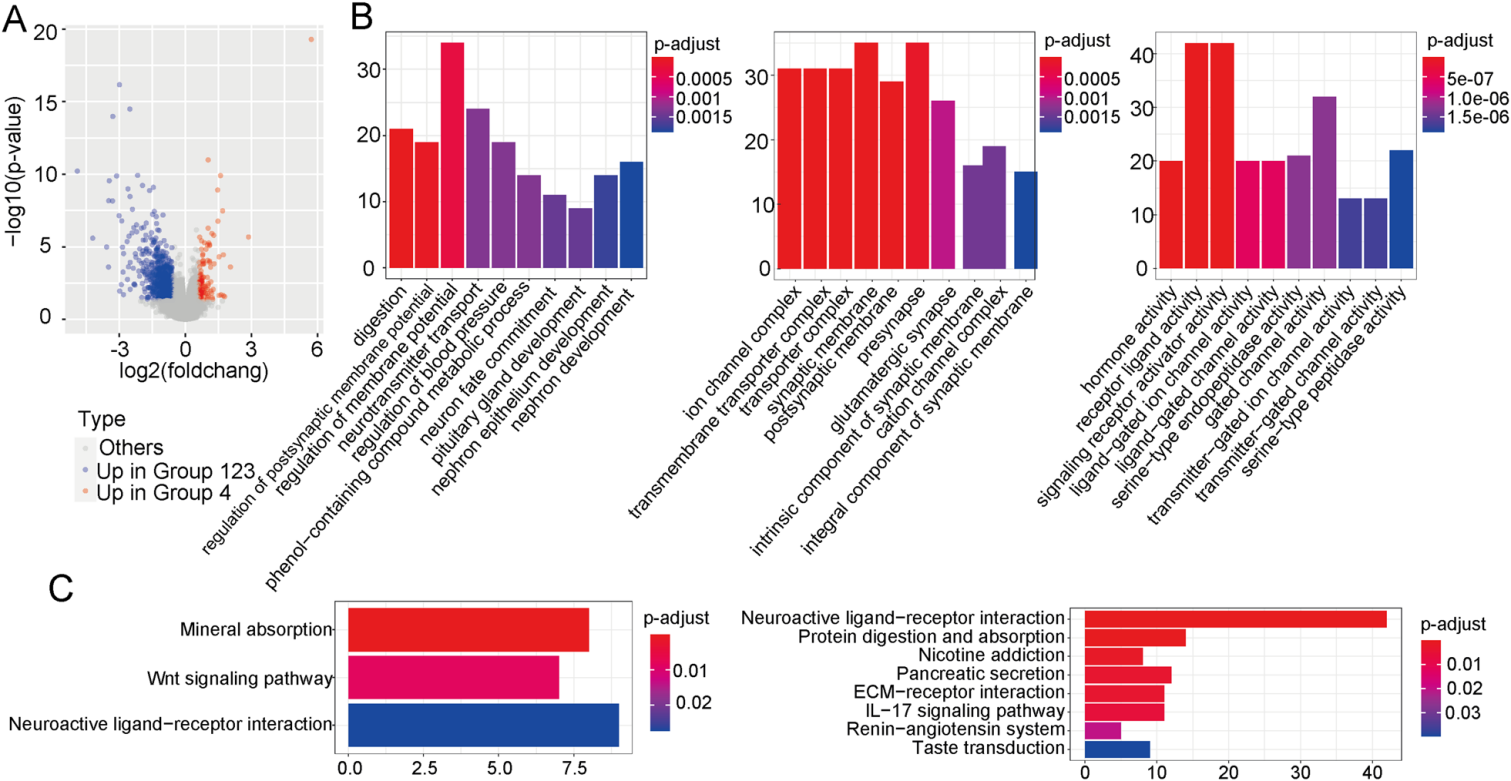
Identification of differentially expressed genes (DEGs) and pathway enrichment analyses in the four subgroups. **a** DEGs in Groups 1–3 and in Group 4. **b** GO analysis indicated that the majority of DEGs (based on different immunotypes) were enriched in cell surface receptor signalling pathways that regulate the immune response and in cell surface receptor signalling pathways that activate the immune response. **c** KEGG pathway analysis showed that the DEGs in Group 4 were enriched in elements of the WNT signalling pathway, whereas the downregulated DEGs were enriched in elements of immune-related response pathways

We used the DESeq package in R software to identify and classify immune system-related DEGs. The upregulated DEGs in Group 4 were positively correlated with the immune classifier-based genes, and the upregulated DEGs in Groups 1, 2, and 3 were negatively correlated with the immune classifier-based genes (Fig. 6A). To further explore the regulatory roles of these DEGs, we used the clusterProfiler package in R software to characterize the associated genes. GO pathway analysis indicated that these DEGs are involved in a variety of cell regulation pathways, including the regulation of membrane potentials, neurotransmitter transport, and those for the regulation of blood pressure. CC analysis indicated that these DEGs play crucial roles in synaptic membranes, the presynapse, the ion channel complex, and transmembrane transporter pathways. MF analysis showed that the regulatory mechanisms for the DEGs are mainly enriched in receptor-ligand activity, signalling-receptor activator activity, and other such pathways (Fig. 6B). KEGG pathway annotations showed that Group 4 was enriched in WNT signalling pathway elements. The downregulated DEGs were enriched in immune response-related pathway elements (Fig. 6C).

**Fig. 7:**
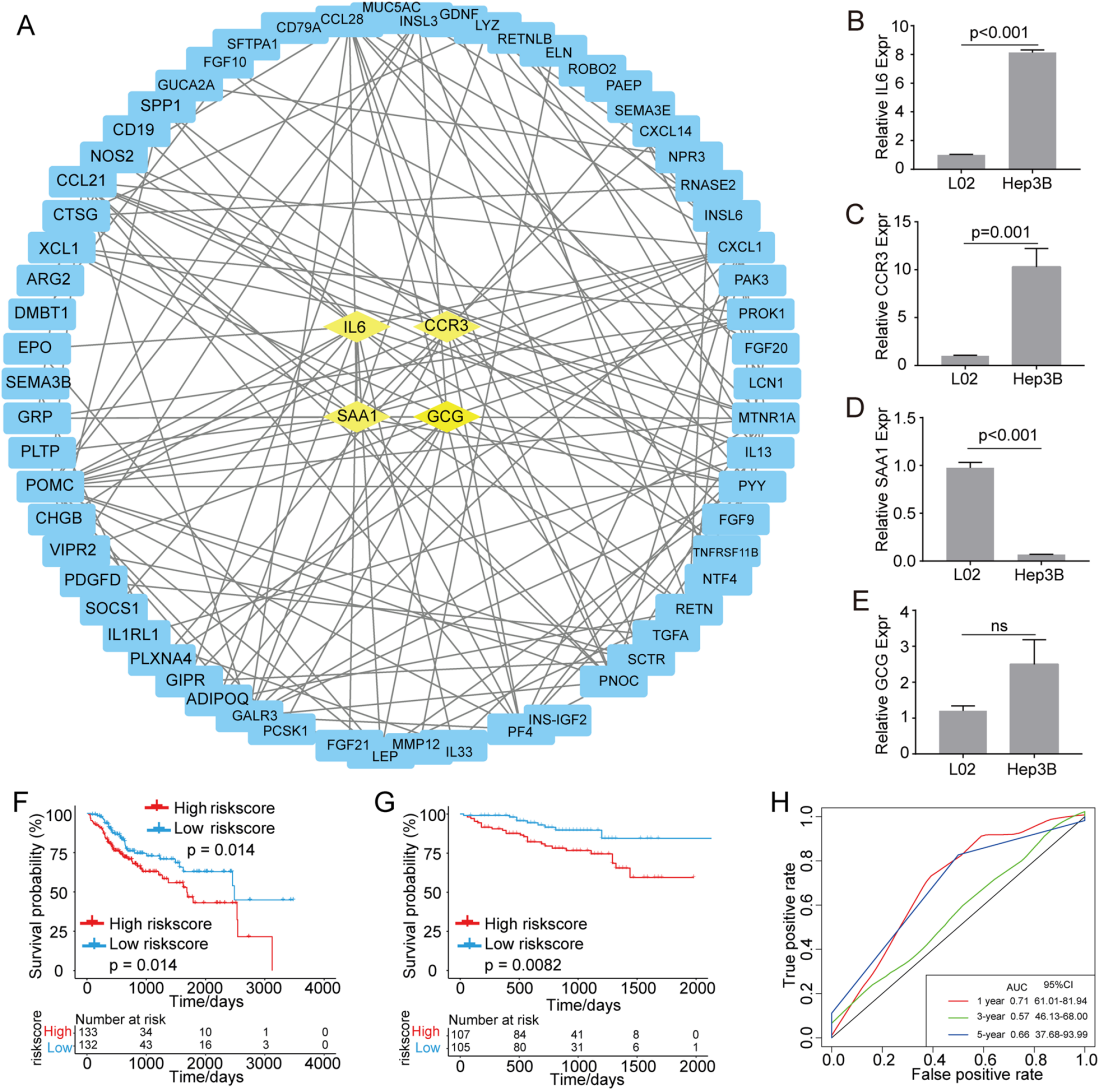
PPI network analysis, the identification of five key immune system-related genes, and a prognostic model based on these five genes. **a** The PPI network analysis revealed four hub genes (IL6, CCR3, SAA1, and GCG) that were closely related to the immune classifier-based DEGs. **b**–**e** qRT-PCR was performed to determine the relative expression of IL6, CCR3, SAA1, and GCG in the HCC cell line (Hep3B) and the control cell line (L02). **f** The key immune system-related genes (*SEMA3A*, *TNFRSF11B*, *GUCA2A*, *SAA1*, and *CALCR*) and the prognostic model based on these genes demonstrated that the group with high risk scores had shorter overall survival times than the group with low risk scores. **g** The biomarkers were validated using the ICGC-LIRI-JP dataset. The five-gene-based prognostic model showed that the group with high risk scores had worse prognoses than the group with low risk scores. **h** Area under the curve (AUC) analyses showed that this predictive model demonstrated good prognostic value

To gain a deeper contextual understanding of their possible interactions, we constructed a PPI network and identified four hub genes (*IL6, CCR3, SAA1*, and *GCG*; Fig. 7A). To further identify HCC prognosis-related genes, Cox regression analyses were performed and yielded 45 DEGs. After applying stringent screening conditions (*p * <  0.01), we identified five immune-related genes that demonstrated significant differences related to prognosis (*SEMA3A*, *TNFRSF11B*, *GUCA2A*, *SAA*, and *CALCR*). In addition, the expression levels of IL6, CCR3, SAA1, and GCG in the HCC cell line (Hep3B) and control cell line (L02) were determined by qRT-PCR. As shown in Fig. 7B, C, the expression of IL6 and CCR3 was higher in the HCC cell line (Hep3B) than in the control cell line (L02). The expression level of SAA1 was lower in Hep3B cells than in L02 cells (Fig. 7D). There was no significant difference in GCG expression between the HCC cell line and the control cell line (Fig. 7E). Using multivariate Cox regression for further analysis, we constructed a risk-coefficient model using these five genes. This model demonstrated that the subgroup with the highest risk scores had poorer prognoses than the subgroup with the lowest risk scores (*p*  =  0.014; Fig. 7F).

To further validate these five immune gene-based biomarkers, Cox regression analyses were applied to datasets from the International Cancer Genome Consortium (ICGC-LIRI-JP). These alternate independent analyses showed that the group with the highest risk scores also had poorer prognoses than the group with the lowest risk scores (Fig. 7G). Area under the curve (AUC) analyses (Fig. 7H), which were used to quantify and evaluate diagnostic accuracy, showed that the AUC value for the five gene-based prognostic model was 0.71 (95% CI  =  61.01–81.94).

### Assessment of immunotherapy responses

**Fig. 8:**
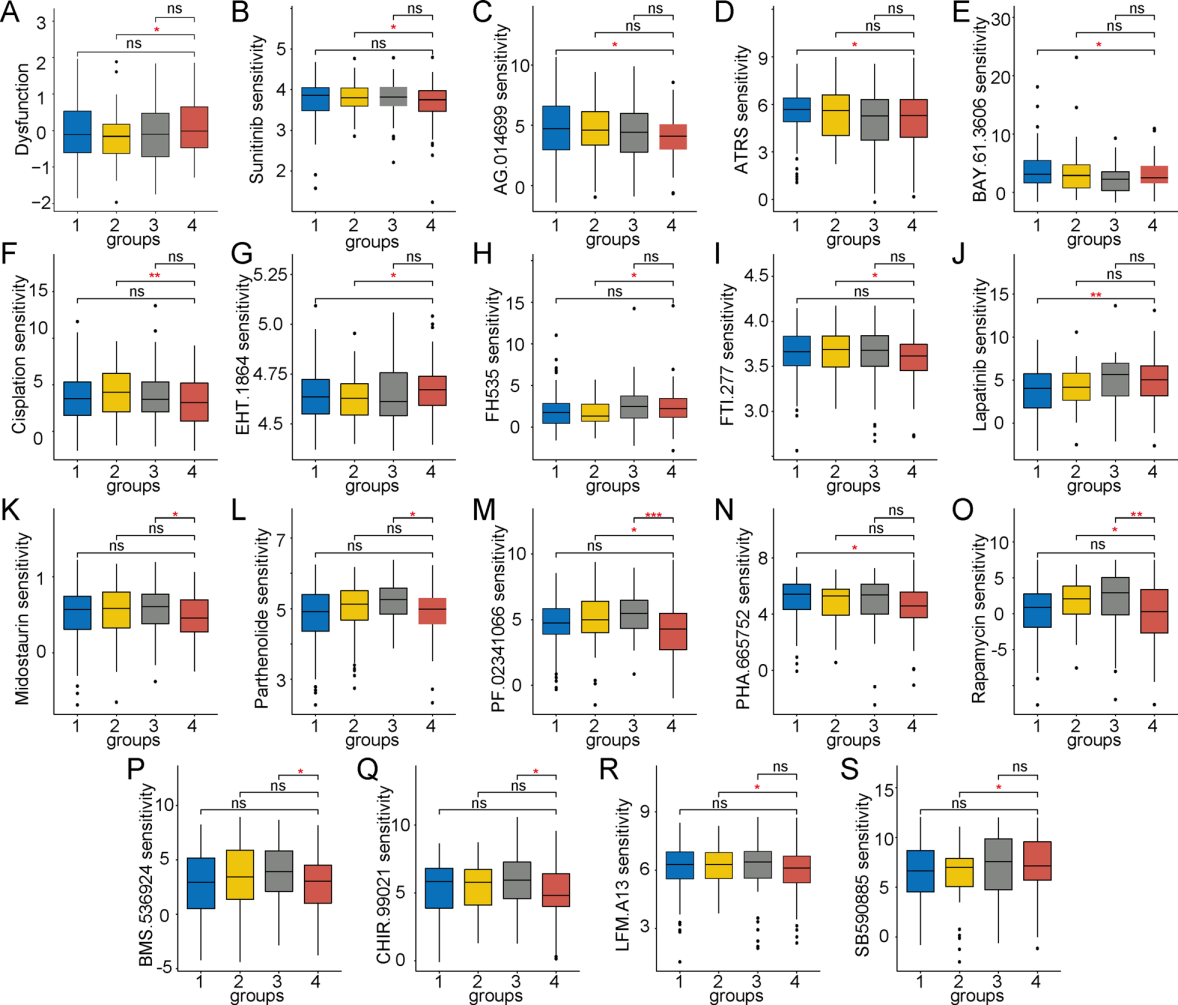
Potential therapeutic drugs for Group 4 based on T cell expression. **a** Group 4 had a higher level of T cell dysfunction than the other subgroups. **b**–**s** Eighteen drugs with obvious differences in sensitivity in Group 4. These results indicate that Group 4 is more sensitive to EHT 1864, FH535, and lapatinib chemotherapy than the other groups

Further studies were performed to explore the relationship between the four immune subgroups and immunotherapy responses by evaluating the clinical immunotherapy responses to PD-1 and CTLA4. Interestingly, Group 4 was characterized as T cell-deficient and had the highest immune deficiency scores (Fig. 8A). These studies confirmed that immune deficiency is involved in tumour immune escape, especially in Group 4.

### Chemotherapy responses

To comprehensively examine the responses of the different immune subgroups to chemotherapeutic agents, we used the pRRophetic algorithm in R software. The results indicated that the half-maximal inhibitory concentration (IC50) values of Group 4 were significantly different than those of the other subgroups (the corresponding drugs are listed in Fig. 8B–S), but not all of these drugs exhibited highly sensitive responses. Only EHT 1864, FH535, and lapatinib exhibited higher sensitivities in Group 4, demonstrating the limited selection of drugs for treating advanced-stage HCC tumours.

## Discussion

HCC is a very aggressive type of cancer with highly heterogeneous malignancies [43]. The tumour microenvironment plays a crucial role in immune cell regulation and cytokine production and plays a central role in cancer development and progression [44]. The emerging field of immunotherapy has increasingly been applied to HCC [14, 43]. However, compared to other cancers, HCC immunotherapy is still in its infancy, and there are still many challenges that need to be addressed, especially for expanding its indications and increasing its benefits [45, 46]. Therefore, personalized immunotherapy strategies and multiple therapy combinations may be required for effective HCC treatment [16, 47]. In the present study, we comprehensively described HCC subgroups based on the expression of immune system-related genes and identified five genes for a prognostic prediction model. A better understanding is still necessary for the criteria required for patient selection and for optimizing any combination strategies to maximize the potential of these approaches.

Previous studies have shown that the liver is a relatively immune-tolerant organ with a high level of immune cell infiltration [48, 49]. Moreover, a variety of immune system regulators are involved in HCC carcinogenesis and progression [14, 50, 51]. To better elucidate the biological functions of HCC immune system-related genes, we identified four groups (Groups 1–4) based on different degrees of immune system gene expression. Based on this classifier, we demonstrated that Group 4 was closely related to the clinical characteristics of advanced HCC, a high level of macrophage infiltration, low immune purity, and poor overall survival. For this type of HCC, the results indicate high levels of immune cell infiltration with few immune cells contributing to anti-tumour functions and activities.

Recently, immunotherapies that have targeted different immune checkpoints have achieved great successes in treating HCC. Such regulation of the immune system facilitates mast cells and activates HLA-G molecules, which are involved in the pathology of chronic hepatitis [52]. HLA molecules are also crucial for T cell-mediated immune surveillance in HCC patients, and HLA-mediated immunomodulatory functions have been observed in numerous pathological conditions, including cancer [35]. HLA-G has shown both prognostic potential and diagnostic value in HCC patients, and HLA molecules have been reported to play crucial roles in the pathogenesis of HCC [53]; therefore, they may be useful as part of an overall immunotherapy strategy against HCC [54]. Accordingly, we found that the subgroups with high immune scores and Group 4 (with high levels of immune cell infiltration) may have better immunotherapy responses.

Furthermore, we assessed the immune classifier and IFN-γ and m^5^C regulators in the different groups. Group 4 exhibited both high IFN-related pathway activation and modifications in m^5^C regulator genes. We also identified four immune system-based hub genes that play crucial roles in immune system-related gene mechanisms that participate as regulators of a variety of gene functions. The five most important immune system DEGs were identified and used as the basis for constructing a prognostic model. This model system was significantly associated with HCC prognoses and may provide better predictions of HCC prognoses when applied clinically.

## Conclusions

The gene expression classifier described in the present study provides a comprehensive understanding of immune system-related genes and HCC characteristics. The resulting prognostic model for predicting HCC outcomes may facilitate the determination of clinically relevant and reliable prognostic indicators for immunotherapy responses.

## Supplementary Information

**Additional file 1: Table S1.** The primers for the qRT-PCR assay.

## Data Availability

The datasets used and/or analysed during the current study are available from the corresponding author upon reasonable request.

## Abbreviations

HCC: Hepatocellular carcinoma
ICB: Immune checkpoint blockade
PD-1: Programmed cell death ligand 1
TIM3: T cell immunoglobulin mucin domain-containing-3
TCGA: The Cancer Genome Atlas
LIHC: Liver hepatocellular carcinoma
ICGC: International Cancer Genome Consortium
ssGSEA: Single-sample gene set enrichment analysis
GSVA: Gene set variation analysis
ESTIMATE: Estimation of STromal and Immune cells in MAlignant Tumours using Expression data
DEGs: Differentially expressed genes
FDR: False discovery rate
FC: Fold change
KEGG: Kyoto encyclopedia of genes and genomes
GO: Gene Ontology
BP: Biological process
MF: Molecular function
CC: Cellular component
PPI: Protein–protein interaction
TIDE: Tumour immune dysfunction and exclusion
ICIs: Immune checkpoint inhibitors
GDSC: Genomics of Drug Sensitivity in Cancer
