# Supplementary material for: Immune classifier-based signatures provide good prognostic stratification and predict the clinical benefits of immune-based therapies for hepatocellular carcinoma
Source: Cancer Cell Int. 2021 Sep 6;21:471. doi: 10.1186/s12935-021-02183-5 (PMC8422634; doi:10.1186/s12935-021-02183-5)
Supplement: Supplementary file 1 — Additional file 1: Table S1. The primers for the qRT-PCR assay. [file 12935_2021_2183_MOESM1_ESM.docx]

**Supplementary Table 1.** The primers for the qRT-PCR assay**.**

| Genes | Primer sequences |
| --- | --- |
| IL6-F | CAATGAGGAGACTTGCCTGGT |
| IL6-R | GGGTCAGGGGTGGTTATTGC |
| CCR3-F | CTCCCTCTGCTCGTTATGGC |
| CCR3-R | GCCACATTGTAGGGTGTCCA |
| SAA1-F | AGAGATTCTTTGGCCATGGTGC |
| SAA1-R | CGCTTTGTATCCCTGCCCTG |
| GCG-F | ATTTCCCAGAAGAGGTCGCC |
| GCG-R | CCCTGGCGGCAAGATTATCA |
